# Compact Design of Annular-Microstrip-Fed mmW Antenna Arrays

**DOI:** 10.3390/s21113695

**Published:** 2021-05-26

**Authors:** Shu-Dong Lin, Shi Pu, Chen Wang, Hai-Yang Ren

**Affiliations:** 1 Hubei Key Laboratory of Roadway Bridge and Structure Engineering, Wuhan University of Technology, Wuhan 430070, China; shudonglin@whut.edu.cn; 2 Department of Physical Science and Technology, School of Science, Wuhan University of Technology, Wuhan 430070, China; renhaiyang@whut.edu.cn; 3 Altair Engineering Software (Shanghai) Co., Ltd., Shanghai 200070, China; chen.wang@altair.com.cn

**Keywords:** microstrip antenna, array, mmW, feeding line, multi-direction

## Abstract

In this paper, a series of four novel microstrip antenna array designs based on different annular-microstrip feeding lines at 60-GHz millimeter wave (mmW) band are proposed, aiming at the potential usage of the mmW coverage antenna with multi-directional property. As the feeding network, the annular contour microstrip lines are employed to connect the patch units so as to form a more compact array. Our first design is to use an outer contour annular microstrip line to connect four-direction linear arrays composed of 1 × 3 rectangular patches, thus the gain of 8.4 dBi and bandwidth of over 300 MHz are obtained. Our second design is to apply the two-direction pitchfork-shaped array each made up of two same linear arrays as the above, therefore the gain of 9.65 dBi and bandwidth of around 250 MHz are achieved. Our third design is to employ dual (inner and outer contour) annular-microstrip feeding lines to interconnect the above four-direction linear arrays, while our fourth design is to bring bridged annular-microstrip feeding lines, both of which can realize the goal of multi-directional radiation characteristic and higher gain of over 10 dBi.

## 1. Introduction

Millimeter wave (mmW) frequency band is shown to have much potential usage not only for the near future wireless communication system like 5G NR FR2, but also for the other upcoming signal coverage system based on the protocols of 802.11ad (Wi-Gig) or 802.11bd. According to previous research, different types of mmW antenna structures working at mmW frequency bands with novel structures or designing methods have been proposed before. The substrate integrated waveguide (SIW) technology, dual-element arrays, and extension structures have been used in designing the directional horn antenna working at 5G mmW bands [1]. Defected ground structure (DGS) [2], 1 × 4 antipodal Vivaldi antenna (AVA) array structure [3], H-shaped and slot-loaded patch with a shorting pin structure [4] have also been proposed in patch antennas working at 5G mmW bands such as 28 GHz. Besides, the rectangular dielectric resonator antenna with circular patch obtains the radiation efficiency at 26 GHz and can be applied in higher frequency of 5G mmW from 24.25 GHz to 27.5 GHz with the optimization of the rectangular slot and cross slot [5]. New structures such as the superposition of identical metallic layers based on metasurface can attain the high gain of 27 dBi at 28 GHz [6]. In the frequency band of 60 GHz, some antennas are combined with IC technology. For example, the low-loss Low Temperature Co-Fired Ceramic (LTCC) has been utilized in wide-band 60 GHz chip antenna and it enables better radiation effectiveness compared with the patch antenna integrated into PCB [7]. A kind of virtual annular antenna has been proposed for use on CMOS IC technology with the achievement of a radiation efficiency of 90% and a gain of 1.43 dBi at about 60 GHz [8]. What is more, different kinds of antennas have been shown such as the inverted-F antenna and quasi-Yagi patch antenna which are working at mmW bands [9]. There are also plenty of designs based on microstrip antenna arrays such as the modified 2 × 2-element and 2 × 2-slot element 60 GHz planar array antenna [10,11], microstrip grid array antennas [12,13], microstrip antenna with comb line shape [14], and a microstrip patch antenna array which excites the frequency selective surface (FSS) with two elements [15], etc. Some of the above-mentioned designs are focusing on achieving the high gain. The microstrip grid array antennas can obtain the 14 dBi-highest-gain at 60 GHz and the impedance bandwidth from 57 GHz to 66 GHz [13]. The microstrip antenna with comb line shape can attain the high gain of 20.3 dBi [14]. Some other methods such as loading Artificial Magnetic Conductor (AMC), constructing composite metamaterial, and isotropic meta-surfaces have been proposed to improve gain [16,17,18]. However, in this paper, our goal is to achieve more homogeneous coverage with more isotropic radiation instead of high gain, while the articles which are focusing on isotropic or multi-directional radiation property are few.

Facing the main issues of obtaining the characteristics of miniaturization, planarity, and integration of antennas and aiming at the potential applications in mmW indoor or outdoor propagation scenarios, a series of four novel microstrip antenna array designs based on different annular-microstrip feeding lines at around 60 GHz are proposed in this paper. In the process of designing the antenna structure to be more compact, the annular microstrip feeding lines which can connect patch units and various compact structures are proposed as the feeding network. On this basis, we improve the surface current distribution of all arrays to be more uniform in order to obtain the multi-directional radiation characteristic under linear polarization mode. Some of our preliminary studies have been already reported in the patents [19,20,21] and conference papers [22,23]. In Section 2, the principle of our design and the two basic designs based on annular microstrip line with different forms of patch arrays will be proposed. In Section 2.2, the fabrication and experimental results of the first basic structure will be shown, which is the verification of our designs. In Section 3, the latter two designs would show the methods of acquiring multi-directional characteristics, with four linear arrays using dual annular feeding method and central feeding mode based on the requirements of uniform surface current distribution.

## 2. Theory and Analysis of Annular Microstrip Feeding Design

### 2.1. Outer-Annular Microstrip-Fed Antenna Array

**Figure 1:**
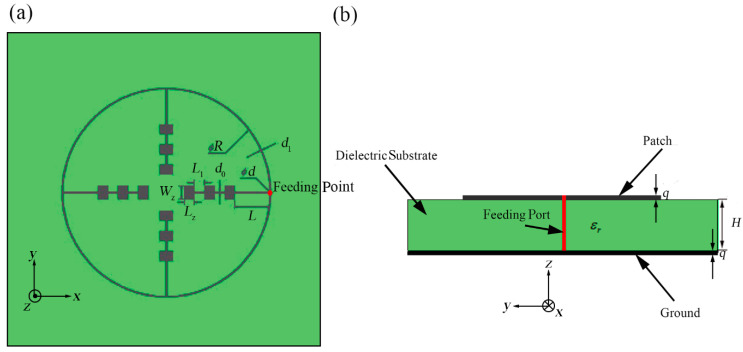
Outer-annular microstrip-fed antenna array, (**a**) top view, (**b**) side view.

To achieve the compact feature, the outer annular microstrip line has been analyzed and designed by us. No matter how many branches there are in each structure, we follow the principle that the length of the outer annular microstrip line between every two branches should be the integral multiple of the half-wavelength of the central frequency. For instance, as shown in Figure 1a, the annular microstrip line is divided into four parts. So, the length of each part of annular microstrip line should be designed as above.

If this requirement has been met, the quantity of the branches can be designed as 2~6 at designer’s option. One point that needs to be assured is that the input impedance of each branch should be equal to 50 Ω after being calculated in parallel. Besides, the initial values of the width of the microstrip lines should generally meet the following computational formulas [24]:

(1) $$m=\frac{Z_{c}\sqrt{2\left(\sqrt{\varepsilon_{r}+1}\right)}}{120}+\frac{\varepsilon_{r}-1}{\varepsilon_{r}+1}\left(0.2258+\frac{0.1208}{\varepsilon_{r}}\right)$$

(2) $$\frac{d_{n}}{q}=\frac{8e^{m}}{e^{2m}-2}$$

where $Z_{c}$ is the impedance of the microstrip line, $\varepsilon_{r}$ is the relative dielectric coefficient, $d_{n}$ (n=0 or n=1) is the width of the microstrip line, and q is the thickness of the microstrip line and m is the interim parameter.

Besides, we choose rectangular patches as the radiation elements in our design. When setting the initial values of the patches, we follow the following computational formulas:

(3) $$\varepsilon_{e}=\frac{\varepsilon_{r}+1}{2}+\frac{\varepsilon_{r}-1}{2}{\left(1+12\frac{H}{L_{z}}\right)}^{-\frac{1}{2}}$$

(4) $$\Delta L=0.412H\frac{\left(\varepsilon_{e}+0.3\right)\left(\frac{L_{z}}{H}+0.264\right)}{\left(\varepsilon_{e}-0.258\right)\left(\frac{L_{z}}{H}+0.8\right)}$$

(5) $$f_{r}=\frac{c}{2\left(W_{z}+2\Delta L\right)\sqrt{\varepsilon_{e}}}$$

where $\varepsilon_{e}$ is the effective relative dielectric coefficient of the substrate, H is the thickness of the dielectric substrate, ∆L is the length of equivalent radiation gap, $f_{r}$ is the resonant frequency, $L_{z}$ is the length of each patch, $W_{z}$ is the width of each patch, and c is the speed of light in vacuum.

The four antenna structures in this paper are flexible and easy to adjust the bandwidth by changing parameters such as the thickness of the dielectric substrate, which means that our designs have good robustness and can meet the general bandwidth requirements of different application scenes. However, employing the equivalent formulas (1)–(5) are not enough to guide us to design the annular-microstrip-fed mmW antenna arrays we need. Hence, all simulations are using a commercial Method of Moments (MoM) solver (Altair Feko) in this paper, which is one of the famous full-wave numerical methods for 3D electromagnetic field computation.

As shown in Figure 1, we propose the outer-annular microstrip-fed antenna array as the basic structure. We use an outer contour annular microstrip line to connect four directional linear arrays with 1 × 3 rectangular patches. We set the white point as the feeding point which is at the center of the right-hand outer annular microstrip line. The punched hole with the diameter of *d* shown in the Figure 1 is designed for the further experimental soldering with the coaxial probe.

All the patches, microstrip line and the ground of bottom layer are applied by silver with the thickness of q= 0.006 mm. Besides, the whole antenna array is etched on both surfaces of the two sides of the aluminum oxide dielectric substrate with the relative dielectric coefficient of $\varepsilon_{r}$ = 9.8 and thickness of H= 0.12 mm [25]. In the simulation, the dielectric substrate is set as infinite plane. Besides, the four identical shaped patch array branches are all designed for implementing the input impedance of 200 Ω in order to match the requirement of input impedance value 50 Ω at the coaxial probe feed port from the observed antenna terminal. The final width of each microstrip line depends on formulas (1) and (2) and the final length of each microstrip line depends on the simulation results. Each patch is designed as working in the central frequency of 60 GHz according to formulas (3)–(5), with the length of 1.05 mm and the width of 0.78 mm.

After analyzing and calculating, we set the inner diameter of the outer annular microstrip line as *R* = 16.50 mm and the width of the outer annular microstrip line as *d*_1_ *=* 0.110 mm. Other detailed parameters of this structure have been demonstrated in Table 1. The meanings of these parameters are shown in Figure 1a.

**Figure 2:**
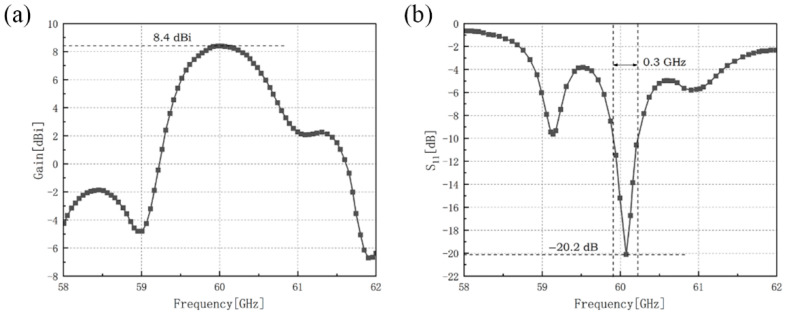
Simulation results of outer-annular microstrip-fed antenna array, (**a**) gain of this antenna array, (**b**) reflection coefficient of this antenna array.

The gain and the reflection coefficient of the outer-annular microstrip-fed antenna array have been shown in Figure 2.

The maximum gain of the outer-annular microstrip-fed antenna array is 8.4 dBi at 60.0 GHz and the minimum reflection coefficient of it is −20.2 dB at 60.1 GHz with the impedance bandwidth of 0.3 GHz. 

**Figure 3:**
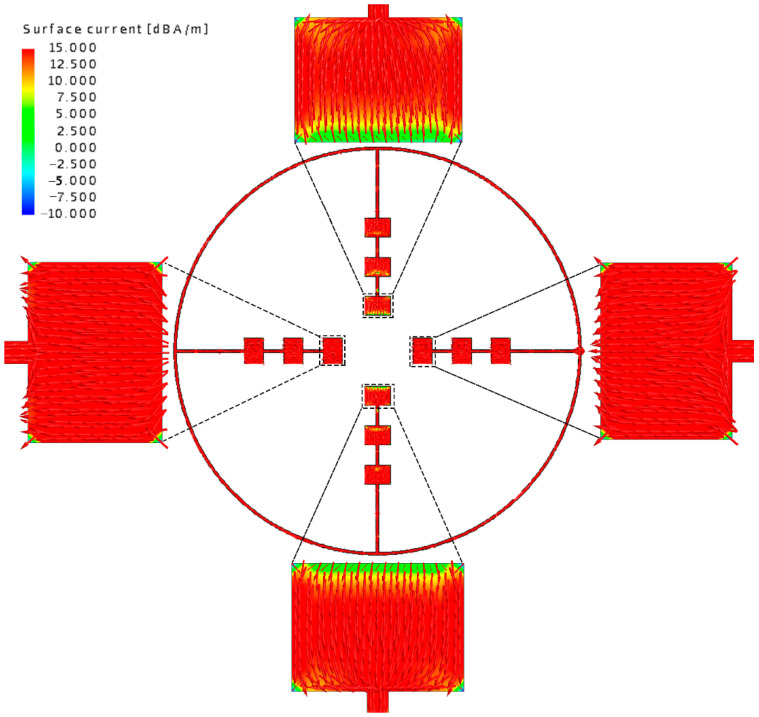
Moduli and the detailed vector plot of the instantaneous current distribution of the outer-annular microstrip-fed antenna array at around 60 GHz.

Moduli and the detailed vector plots of the instantaneous surface current distribution of the outer-annular microstrip-fed antenna array are shown in Figure 3.

**Figure 4:**
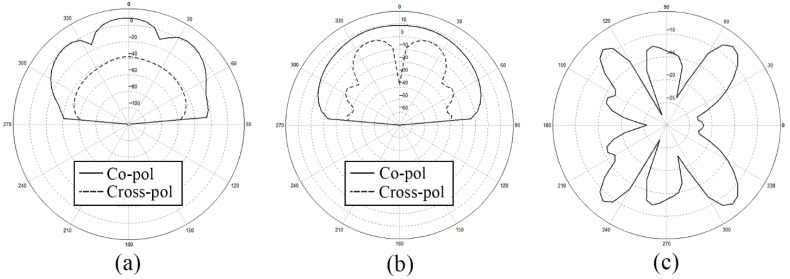
Radiation pattern of the outer-annular microstrip-fed antenna array (**a**) *x-z* plane, (**b**) *y-z* plane, (**c**) *x-y* plane.

After analyzing the surface current distribution in Figure 3, we find that the edge feeding mode will generally cause the currents on the side feeding units too little. This is the reason why the radiation range of antenna is relatively narrow and non-uniform. What is more, looking at the detailed vector plot of the surface current on patches, it is shown that patches on the parallel main feeding units are the same in phase while patches on the side feeding units are in opposition of phase. The opposition of phase of the opposite branches will influence the gain and the aperture efficiency of the antenna array to some extent. We also show the *x-z* plane, *y-z* plane, and *x-y* plane of the radiation pattern of the outer-annular microstrip-fed antenna array in Figure 4. The pure line represents the co-polarization, whereas the dashed line represents the cross-polarization pattern.

### 2.2. Verification of the Basic Structure of Outer-Annular Microstrip-Fed Antenna Array

#### 2.2.1. Comparation of Simulation Result and Experimental Result

**Figure 5:**
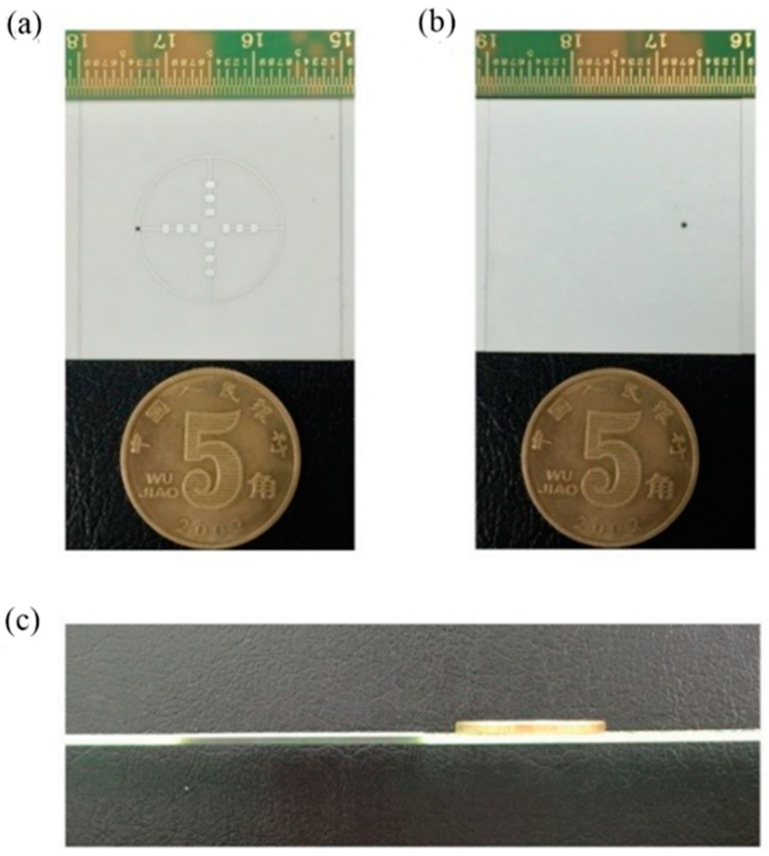
Photo of the manufactured object of the first structure, (**a**) the top view, (**b**) the bottom view, (**c**) the side view.

**Figure 6:**
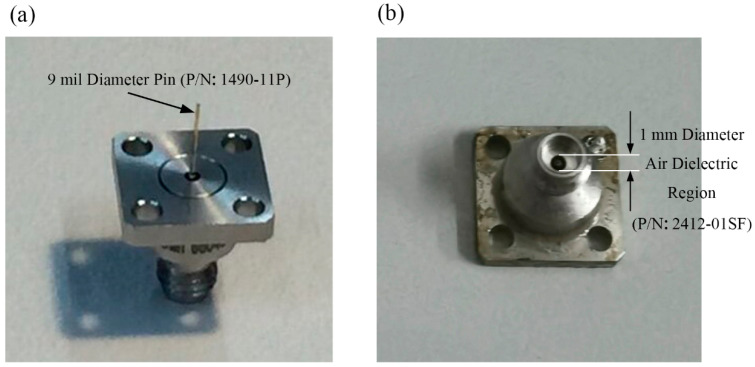
Photo of the 1 mm connector, (**a**) 3D view from pin to thread, (**b**) 3D view from thread to pin.

**Figure 7:**
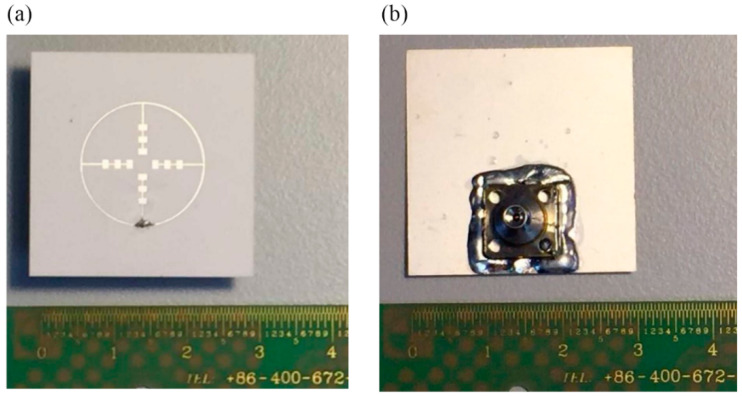
Photo of the manufactured object after being soldered, (**a**) the top view, (**b**) the bottom view.

In the verification, we choose to make the first basic structure into manufactured object. The metal patch was printed on an aluminum oxide substrate with $\varepsilon_{r\;}=9.8$ and H=0.12 mm, as depicted in Figure 1. The patch is made of silver with the thickness of q=0.006 mm. The antenna adopts the coaxial feeding port, and the feeding point is located as Figure 1 shown. The antenna is connected to the vector network analyzer by using a 1 mm connector from Southwest Microwave, Inc. The manufactured object is shown in Figure 5. While hermetically packaging, the coaxial connector is attached to the threaded mounting holes where a glass bead is positioned mid-way between them. The 1 mm connector is shown in Figure 6 and the manufactured object after soldering is shown in Figure 7. The connector is soldered on the bottom side of the ground plane by flange as shown in Figure 7b.

**Figure 8:**
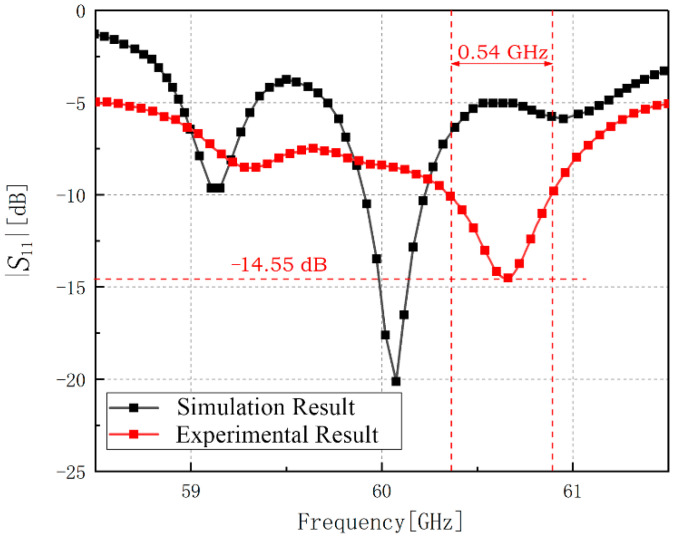
Simulation result and experimental result of the reflection coefficient of the first structure.

The experimental work was carried out in Rohde & Schwarz ^®^ open laboratory in Shanghai with the ZVA110 vector network analyzer (10 MHz—110 GHz). The results are shown in Figure 8. The resonant frequency of the antenna is 60.65 GHz and the minimum reflection coefficient of it is −14.55 dB at 60.65 GHz with the bandwidth of 0.54 GHz. Because the antenna structure in this paper is easily affected by the 1 mm connector structure, the antenna’s resonant frequency experimental result has shifted to a certain extent with the similar entire trend compared with the simulation result.

#### 2.2.2. Add-on Analysis with a Connector

**Figure 9:**
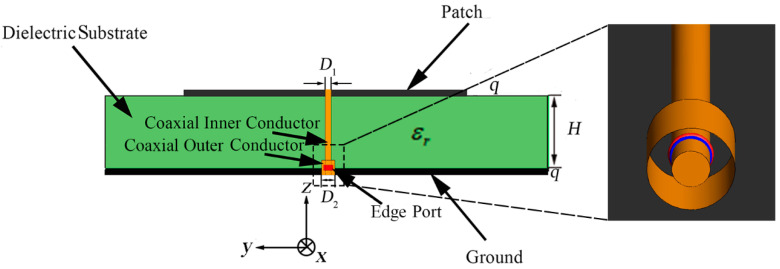
The side view and local detail of the verification model with coaxial feeding method.

**Figure 10:**
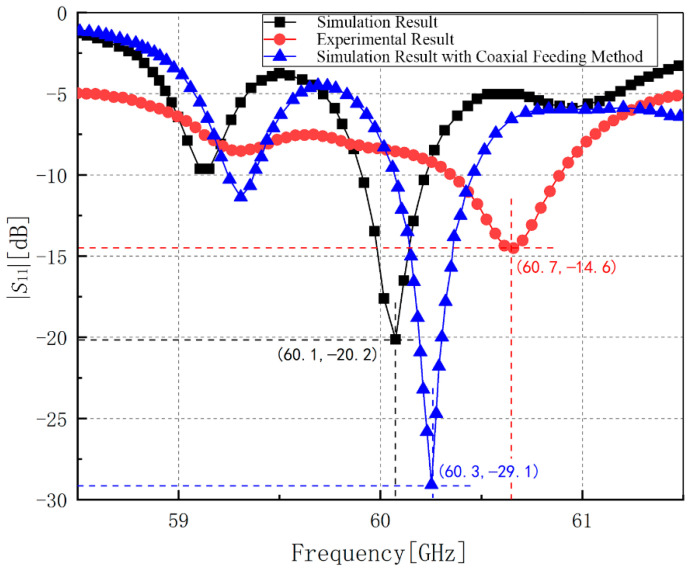
Comparation of the simulation result, experimental result, and the simulation result with coaxial feeding method.

In the simulation, we set the feeding port as the wire port which is more consistent with the actual application scenario. However, in order to achieve a more precise experimental result, the coaxial feeding method is used in the experiment. So, the possible cause of the shift of the result is the 1 mm connector. Aiming at this point, we still use Altair FEKO to analyze the characteristics of the antenna under the coaxial feeding method. The verification model with coaxial feeding method is shown in Figure 9. We set the material of the coaxial feeding conductors as perfect electric conductor. The coaxial feeding port is composed of internal cylinder block and outer cylindrical surface. The internal diameter is $D_{1}=0.005$ mm and the outer diameter is $D_{2}=0.0146$ mm. The coaxial feeding method penetrates the dielectric substrate to feed the antenna array. It is worth mentioning that the coaxial feeding method with edge port in the simulation is just a kind of approximation of the 1 mm connector because of the high complexity of the 1 mm connector. The comparation of the simulation result, experimental result, and the simulation result with coaxial feeding method is shown in Figure 10.

As shown in Figure 10, after using the coaxial feeding method with edge port to replace the original wire feeding port, the resonance frequency moves 0.2 GHz to the right which has a similar trend as the experimental result. Additionally, the bandwidth appears to overlap with the experimental result, which means the results are almost coincident. It is necessary to state that various probable factors will influence the experimental result and enlarge the difference of resonant frequency in the experimental process, especially in such a high frequency band, such as the poor soldering, the change of the resistance because of the soldering tin, and the size of the dielectric substrate. Besides, the resonant frequency of our experimental result is still in the 60-GHz frequency band and the shift is far less than the possible deviation because of the manufacture craft and test process which means it makes no sense in focusing on such deviation in practical application. Thus, our experimental result can be verified.

### 2.3. Two-Direction Pitchfork-Shaped Microstrip Antenna Array

**Figure 11:**
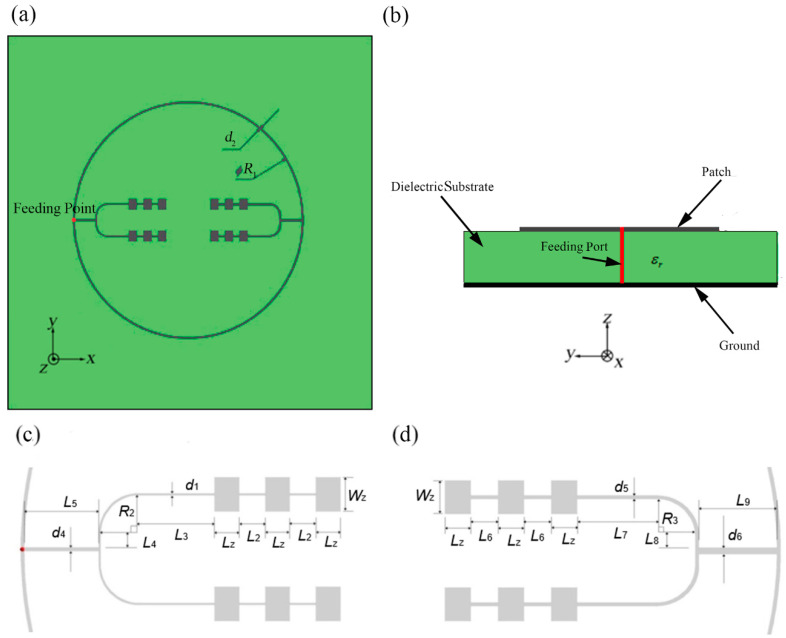
Two-direction pitchfork-shaped microstrip antenna array, (**a**) top view, (**b**) side view, (**c**) the left-side pitchfork-shaped branch (input impedance of 150 Ω), (**d**) the right-side pitchfork-shaped branch (input impedance of 75 Ω).

As shown in Figure 11, we propose the microstrip antenna array consists of outer annular microstrip line and two pitchfork-shaped array branches as the second design. Each pitchfork-shaped branch is composed by two linear arrays each with 1 × 3 rectangular patches. This design is similar to the basic design, but the difference is that we use pitchfork-shaped feeding mode inside the outer annular microstrip line. Initially, the structure was still designed as four array branches. However, the upper and lower branches will influence the radiation characteristics because of the opposition of phase of the two side feeding units. So, we finally adopt two opposite array branches.

We set the red point as the feeding point which is at the center of the left-hand outer annular microstrip line in order to feed the two asymmetrical radiating pitchfork-shaped branches. The two pitchfork-shaped patch array branches consist of twelve rectangular patches in total.

The materials and thicknesses of patches, microstrip line, and the mental ground of bottom layer are the same as normal annular-shaped microstrip antenna array. Besides, in order to meet the requirement of the input impedance value 50 Ω at the coaxial probe feed port from the observed antenna terminal, the two pitchfork-shaped branches need to be designed for implementing the input impedance of 75 Ω and 150 Ω, respectively.

We set the inner diameter of the outer annular microstrip line as $R_{1}$ = 25.870 mm and the width of the outer annular microstrip line as $d_{2}\;$*=* 0.110 mm, as shown in Figure 9. Other detailed parameters of the structure have been demonstrated in Table 2. The meanings of these parameters are shown in Figure 9.

**Figure 12:**
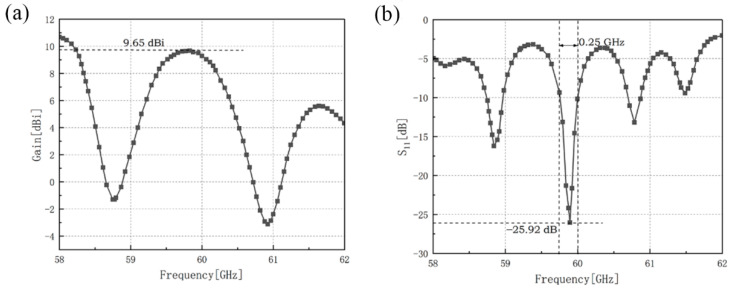
Simulation results of two-directional pitchfork-shaped microstrip antenna array, (**a**) gain of this antenna array, (**b**) reflection coefficient of this antenna array.

The gain and the reflection coefficient of the microstrip antenna array consists of outer annular microstrip line and two pitchfork-shaped array branches have been shown in Figure 12.

The maximum gain of two-direction pitchfork-shaped microstrip antenna array is 9.65 dBi at 59.8 GHz and the minimum reflection coefficient of it is −25.92 dB at 59.9 GHz with the impedance bandwidth of 0.25 GHz. Our designs reach a good impedance matching. 

**Figure 13:**
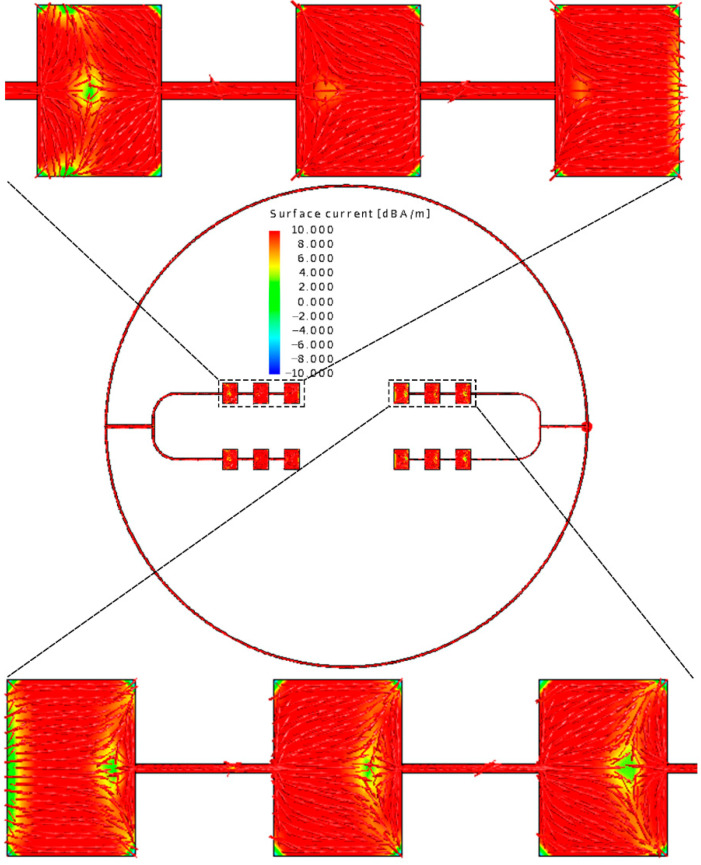
Moduli and the detailed vector plot of the instantaneous current distribution of two-direction pitchfork-shaped microstrip antenna array at around 60 GHz.

Moduli and the detailed vector plots of the instantaneous surface current distribution of two-direction pitchfork-shaped microstrip antenna array are shown in Figure 13. Without the side feeding units, the only opposite units are the same in phase which relatively increase the gain and the aperture efficiency of the antenna array compared with the first structure. The asymmetric design will impact the current on the branch with larger input impendence relatively little. This will also mean that antenna cannot obtain uniform radiation on more directions.

**Figure 14:**
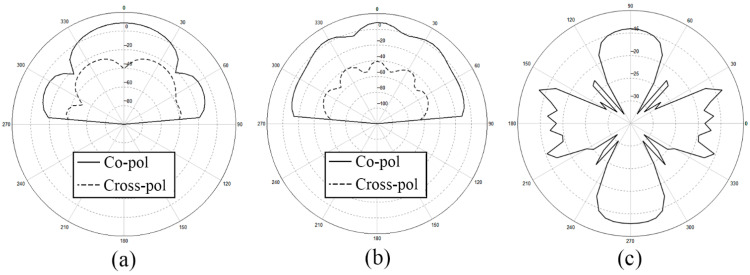
Radiation pattern of two-direction pitchfork-shaped microstrip antenna array (**a**) *x-z* plane, (**b**) *y-z* plane, (**c**) *x-y* plane.

Besides, we show the *x-z* plane, *y-z* plane, and *x-y* plane of the radiation pattern of pitchfork-shaped microstrip antenna array in Figure 14.

## 3. Improvement on Multi-Directional Design

In this part, two solutions of acquiring the characteristic of more uniform multi-directional radiation on the basis of outer annular microstrip line will be shown. More uniform multi-directional radiation does not mean better radiation characteristic but would probably be used in different usage scenarios.

### 3.1. Dual Annular-Microstrip-Fed Antenna Array

**Figure 15:**
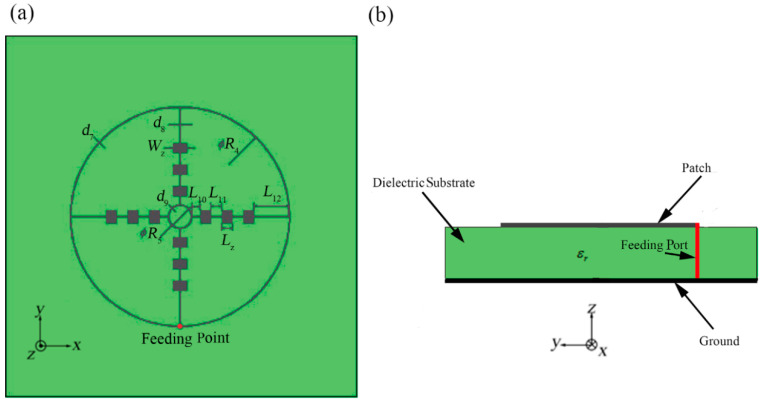
The dual-annular microstrip-fed antenna array, (**a**) top view, (**b**) side view.

This structure is shown in Figure 15 as the third design. The main purpose of this design is obtaining the characteristic of more uniform multi-direction radiation. In the first two designs, we can find that the edge feeding mode will generally cause the current on the non-main feeding units which are not connected with the feeding point is too small, which leads to the problem of inhomogeneous circumferential radiation. Aiming at solving this disadvantage, our researchers try to optimize the surface current distribution of the arrays to be uniform. In this case, in order to attain the characteristic of multi-directional radiation, it is also important to maintain more directional linear arrays. Our thought is to achieve this goal under the same edge feeding mode. Initially, we attempt to use the simple cruciform microstrip lines to interconnect the four linear arrays. However, because the length of microstrip lines in this situation is hard to be designed precisely enough, the impedance matching cannot achieve a favorable consequence. So, compared with pitchfork-shaped microstrip antenna array, in this design, we employ dual (inner and outer contour) annular microstrip lines to interconnect the four directional linear arrays with 1 × 3 rectangular patches. The inner annular microstrip line will connect the other end of the four branches which effectively make the current distribution on the four branches tend to be uniform. The design method of the inner annular microstrip line is the same as it of the outer annular microstrip line which means the length of the inner annular microstrip line between every two branches should also be the integral multiple of the half-wavelength of the central frequency. What is more, in order to ensure normal current feeding of the inner annular microstrip line, the width of the inner annular microstrip line has to be the same as other adjacent microstrip lines.

After analyzing and optimizing, we determine the values of the parameters which are shown in Table 3. The meanings of the parameters are shown in Figure 15.

**Figure 16:**
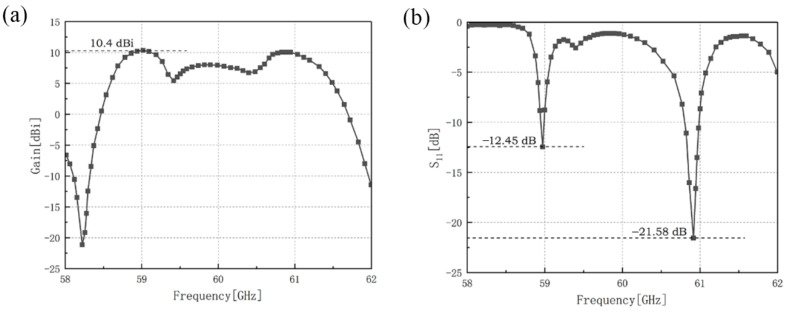
Simulation results of dual-annular microstrip-fed antenna array, (**a**) gain of this antenna array, (**b**) reflection coefficient of this antenna array.

The gain and the reflection coefficient of dual-annular microstrip-fed antenna array of microstrip antenna array is shown in Figure 16.

The results show that the maximum gain of dual-annular microstrip-fed antenna array of microstrip antenna array is 10.4 dBi at 59.0 GHz. In the frequency band of 59.0 GHz–61.0 GHz, this antenna can remain a relatively high gain. Besides, the minimum reflection coefficient of it is −21.58 dB at 60.9 GHz and the reflection coefficient near the frequency of 58.97 GHz can also attain below −10 dB. 

**Figure 17:**
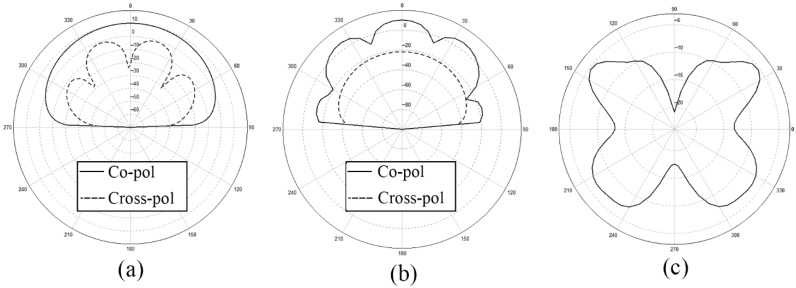
Radiation pattern of dual-annular microstrip-fed antenna array (**a**) *x-z* plane, (**b**) *y-z* plane, (**c**) *x-y* plane.

For further confirmation, we depict the *x-z* plane, *y-z* plane, and *x-y* plane of the radiation pattern of dual-annular microstrip-fed antenna array in Figure 17. 

### 3.2. Bridged Annular-Microstrip-Fed Antenna Array

**Figure 18:**
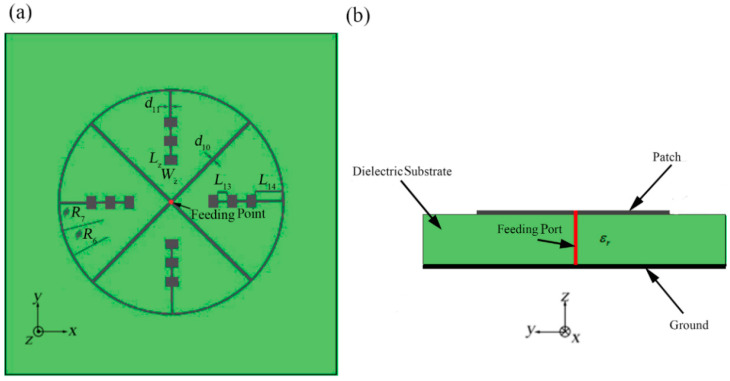
Bridged annular-microstrip-fed antenna array, (**a**), top view, (**b**), side view.

This structure is shown in Figure 18. This design based on a bridged annular microstrip feeding line for the same four directional linear arrays with 1 × 3 rectangular patches as above structure. It proposes another solution for acquiring the characteristics of more uniform multi-directional radiation. The difference from the above three structures is that this structure uses central feeding mode. In addition to the outer microstrip line, this structure also contains mutually perpendicular straight internal microstrip lines with the same quantity as the array branches. One end of each internal microstrip line is connected to the ring midpoint of the connection point between the adjacent array unit and the outer ring microstrip line and the other end is at the center of overall structure. The internal microstrip lines are designed to match the central feeding mode which means each internal microstrip line should also implement the input impedance of 200 Ω. The length of each internal microstrip line should also be the integral multiple of the half-wavelength of the central frequency. With the central feeding mode, the structure has a high symmetry.

In order to gain a better simulation result, we set all the patches, microstrip line, and the mental ground of bottom layer as copper with the thickness of 0.006 mm which are different from the above three structures. However, the structure and material of the dielectric substrate are the same.

Because of the difference of the feeding mode, some structural parameters will have a relatively larger adjustment. The values of the parameters are shown in Table 4. The meanings of the parameters are shown in Figure 18.

**Figure 19:**
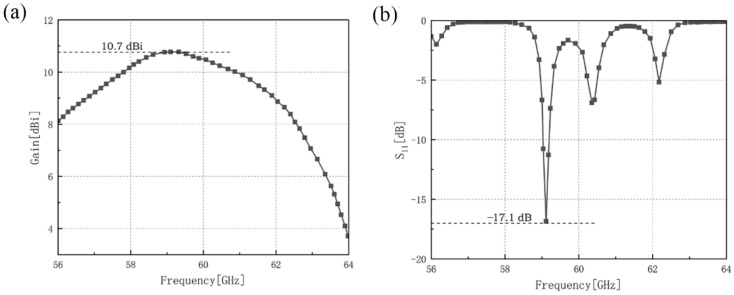
Simulation results of bridged annular-microstrip-fed antenna array, (**a**) gain of this antenna array, (**b**) reflection coefficient of this antenna array.

The gain and the reflection coefficient of bridged annular-microstrip-fed antenna array is shown in Figure 19.

The maximum gain of bridged annular-microstrip-fed antenna array of microstrip antenna array is 10.7 dBi at 59.0 GHz and the minimum reflection coefficient of it is −17.1 dB at 59.1 GHz. Our designs reach a good impedance matching.

**Figure 20:**
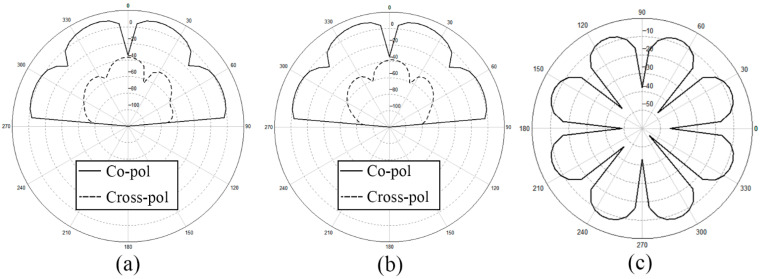
Radiation pattern of bridged annular-microstrip-fed antenna array (**a**) *x-z* plane, (**b**) *y-z* plane, (**c**) *x-y* plane.

We depict the *x-z* plane, *y-z* plane, and *x-y* plane of the radiation pattern of bridged annular-microstrip-fed antenna array in Figure 18. With relatively uniform multi-directional radiation *H*-plane patterns shown in Figure 17 and Figure 20, the latter two designs are multi-directional radiation antennas and have wide radiation ranges which achieve our goal.

## 4. Conclusions

In this paper, aiming at the wider employment of the mmW frequency band in the future, we propose four novel structures of microstrip antenna array working at 60-GHz waveband in total. Arrays are all composed of outer annular microstrip line and varying quantities and shapes of patch array branches. The quantity of the branches can be designed as 2~6 at designer’s option. All the feeding units are contained in the space inside the outer annular microstrip line, in order to obtain a more compact structure. The former two designs are the basic designs which show our design principle. The latter two designs are proposed to obtain the multi-directional radiation feature with the methods of dual annular-microstrip feeding lines and central feeding mode. After simulating and optimizing, we finally achieve the goal of miniaturization of antenna and maintaining symmetrical high-gain radiation in multiple directions under the condition of good impedance matching. Our study can make up for the deficiency of multi-direction characteristics in previous or current research on the mmW microstrip antenna array. In addition, we carried out the physical production and experimental verification of the first basic structure. We obtained the results of the minimum reflection coefficient of −14.55 dB at 60.65 GHz with the bandwidth of 540 MHz, which achieve a good match with the simulation results, so as to prove the correctness of our design. We anticipate that the design of this novel category of structures can provide potential guidelines for the future mmW coverage antenna design or practice.

## Figures and Tables

**Table 1:** Parameters of outer-annular microstrip-fed antenna array.

Parameter	Value (mm)	Parameter	Value (mm)
*W*_z_	1.050	*L*_1_	0.830
*L*_z_	0.780	*d*_1_	0.110
*d*_0_	0.130	*R*	16.500
*L*	2.830	*d*	0.800
*q*	0.006	*H*	0.12

**Table 2:** Parameters of two-side pitchfork-shaped branches.

Parameter	Value (mm)	Parameter	Value (mm)
*L*_z_	0.780	*L*_5_ *, L*_9_	2.445
*L*_2_ *, L*_6_	0.860	*W*_z_	1.050
*L*_3_ *, L*_7_	2.490	*d*_2_	0.11
*L*_4_	0.445	*d*_3_ *, d*_5_	0.055
*L*_8_	0.390	*d*_4_	0.110
*R*_1_	25.870	*d*_6_	0.220
*R*_2_	1.243	*R*_3_	1.215

**Table 3:** Parameters of dual-annular microstrip-fed antenna array.

Parameter	*d*_7_, *d*_9_	*d*_8_	*W*_z_	*R*_4_	*L*_10_	*L*_11_	*L*_12_	*L_z_*	*R*_5_
**value (mm)**	0.130	0.130	1.050	16.180	0.580	0.830	2.580	0.780	1.590

**Table 4:** Parameters of bridged annular-microstrip-fed antenna array.

Parameter	Value (mm)	Parameter	Value (mm)
R_7_	19.21	*W_z_*	1.050
*R*_6_	18.95	*L_z_*	0.780
*d*_10_	0.226	*L*_13_	0.830
*d*_11_	0.130	*L*_14_	2.580

## Data Availability

Not applicable.
